# Bacterial Respiration and Growth Rates Affect the Feeding Preferences, Brood Size and Lifespan of *Caenorhabditis elegans*

**DOI:** 10.1371/journal.pone.0134401

**Published:** 2015-07-29

**Authors:** Li Yu, Xiaomei Yan, Chenglong Ye, Haiyan Zhao, Xiaoyun Chen, Feng Hu, Huixin Li

**Affiliations:** 1 Soil Ecology Lab, College of Resources and Environmental Science, Nanjing Agricultural University, Nanjing, 210095, People’s Republic of China; 2 College of Resources and Environmental Science, Nanjing Agricultural University, Nanjing, 210095, People’s Republic of China; Chinese Academy of Sciences, CHINA

## Abstract

Bacteria serve as live food and nutrients for bacterial-feeding nematodes (BFNs) in soils, and influence nematodes behavior and physiology through their metabolism. Five bacterial taxa (*Bacillus amyloliquefaciens* JX1, *Variovorax* sp. JX14, *Bacillus megaterium* JX15, *Pseudomonas fluorescens* Y1 and *Escherichia coli* OP50) and the typical BFN *Caenorhabditis elegans* were selected to study the effects of bacterial respiration and growth rates on the feeding preferences, brood size and lifespan of nematodes. *P*. *fluorescens* Y1 and *E*. *coli* OP50 were found to be more active, with high respiration and rapid growth, whereas *B*. *amyloliquefaciens* JX1 and *B*. *megaterium* JX15 were inactive. The nematode *C*. *elegans* preferred active *P*. *fluorescens* Y1 and *E*. *coli* OP50 obviously. Furthermore, worms that fed on these two active bacteria produced more offspring but had shorter lifespan, while inactive and less preferred bacteria had increased nematodes lifespan and decreased the brood size. Based on these results, we propose that the bacterial activity may influence the behavior and life traits of *C*. *elegans* in the following ways: (1) active bacteria reproduce rapidly and emit high levels of CO_2_ attracting *C*. *elegans*; (2) these active bacteria use more resources in the nematodes’ gut to sustain their survival and reproduction, thereby reducing the worm's lifespan; (3) inactive bacteria may provide less food for worms than active bacteria, thus increasing nematodes lifespan but decreasing their fertility. Nematodes generally require a balance between their preferred foods and beneficial foods, only preferred food may not be beneficial for nematodes.

## Introduction

Diet provides carbohydrates, fats, proteins and other nutrients to support the growth and daily activities of animals [1]. Diet also influences an animal's behavior and life traits such as fecundity and aging. Bacteria are food for bacterial-feeding nematodes (BFNs) in soils, and BFNs require numerous bacteria to sustain growth, as an individual worm requires as many as 10^3^−10^5^ bacteria per day [2]. Therefore, bacterial quality and metabolism play important roles in regulating BFNs feeding behavior and life traits.

In nature, animals must make decisions regarding what to eat [3]. The ability to locate and access food is critical for survival and reproduction. BFNs encounter many types of bacteria in soils ranging from beneficial to harmful. They should therefore evaluate their prey before making a feeding choice. In the lab, BFNs exhibit distinct feeding preferences for different bacteria [4–6]. For example, Salinas et al. [5] found that *Cephalobus brevicauda* strongly prefers gram-negative and small-celled bacteria. Shtonda and Avery [7] concluded that *C*. *elegans* searches for high quality food that best supports its growth and avoids unfavorable bacteria. Some experimental evidence has shown that *C*. *elegans* can distinguish between pathogenic and nonpathogenic bacteria and avoid the former [8–10]. However, other factors that affect BFNs feeding choices remain unclear. Many insect species use CO_2_ as an olfactory signal to locate food or hosts [11]; therefore, we hypothesize that the concentration of CO_2_ released through bacterial respiration could influence BFNs feeding behavior.

Bacteria can not only influence the feeding behavior of BFNs, but also regulate nematodes lifespan and brood size according to bacteria availability [12,13]. Lifespan and fertility are recognized as basic life traits of organisms and diet has significant effects on organismal lifespan and fertility [14–16]. *C*. *elegans* fed *E*. *coli* OP50 were found to have shorter lifespan than those fed *E*. *coli* HT115 [17]. MacNeil et al. [13] found that *C*. *elegans* fed the soil bacteria *Comamonas* DA1877 had lower reproduction and shorter longevity than worms fed *E*. *coli* OP50. In another study, the preferred bacteria of *Cephalobus brevicauda* resulted in the lowest reproduction of nematodes [5]. In addition, Coolon et al. [18] found that *C*. *elegans* preferred *Pseudomonas* sp. over *B*. *megaterium* but that worms lived longer on *B*. *megaterium* than on *Pseudomonas* sp. As bacteria are a live food, we hypothesize that bacterial activity may have direct or indirect effects on BFNs lifespan and brood size.

The type, size, pathogenicity and toxicity of bacteria can all affect BFNs. Moreover, bacterial respiration and growth rates can also affect BFNs. Some researchers have focused on the effects of bacterial respiration on the lifespan of *C*. *elegans* [19]. Bacterial respiration may be an indicator of bacterial activity and bacterial population size and thereby influence BFNs behavior and life traits. Bacteria respire to release CO_2_, which could influence BFNs sensitivity, and bacterial respiratory strength also affects their ability to colonize in the BFNs guts [19]. In this work, we selected five bacterial species (*Bacillus amyloliquefaciens* JX1, *Variovorax* sp. JX14, *Bacillus megaterium* JX15, *Pseudomonas fluorescens* Y1 and *Escherichia coli* OP50) and the typical model BFN *C*. *elegans* for experimentation. We aimed to (1) determine the feeding preferences of *C*. *elegans* and the effects of bacterial respiration and growth rates on nematode feeding preferences; (2) evaluate the effects of bacteria on the lifespan and brood size of *C*. *elegans*; (3) explore the relationships between bacterial activity and *C*. *elegans* feeding preferences, lifespan and brood size.

## Materials and Methods

### Preparation of bacteria

Soil bacteria were obtained from a sandy loam alluvial soil collected from Banqiao Town, Nanjing City, Jiangsu Province, China. No specific permissions were required for the described field studies. We confirmed that the location of the soil collection was not privately owned or protected in any way, and the field studies did not involve endangered or protected species. The bacteria were obtained by plating a serial dilution of soil suspension onto LB medium dishes (10 g L^-1^ tryptone, 5 g L^-1^ yeast extract, 10 g L^-1^ NaCl, and 17 g L^-1^ agar, pH 7.0); then, the bacteria were purified by picking up the single colony onto LB medium. Finally, we selected 4 representative species of bacteria for our experiments. All of these species were sequenced by 16S rRNA genes (Table 1). Additionally, the four bacteria, along with *E*. *coli* OP50 obtained from the CGC (Caenorhabditis Genetics Center, USA), were prepared in liquid LB medium freshly at a shaking speed of 180 rpm, and the bacterial OD_600_ values were normalized to 1.

**Table 1:** Descriptions of the bacteria used for the experiments.

Bacteria	Shape	Gram stain	Accession number
*B*. *amyloliquefaciens* JX1	rod	G+	JX424611
*Variovorax* sp. JX14	rod	G-	JX424612
*B*. *megaterium* JX15	rod	G+	JX424613
*P*. *fluorescens* Y1	rod	G-	KC962432
*E*. *coli* OP50	rod	G-	-

### Preparation of nematodes

*Caenorhabditis elegans* N2 obtained from the CGC were cultivated on freshly prepared nematode growth medium (NGM) (3 g NaCl, 2.5 g peptone, 17 g agar and 975 ml H_2_O, autoclaved; cooled to 55°C, further added 1 ml 1 M CaCl_2_, 1 ml 5 mg/ml cholesterol in ethanol, 1 ml 1 M MgSO_4_ and 25 ml 1 M KPO_4_ buffer, and filtered through 0.22 μm filter). Petri plates seeded with *E*. *coli* OP50, a standard bacterial food source for *C*. *elegans* in the lab [20]. Any petri plates contaminated with fungi or other bacteria should be discarded.

The worms were cultured at 20°C for 10 days, rinsed with M9 buffer (5 g NaCl, 3 g KH_2_PO_4_, 6 g Na_2_HPO_4_, 1 mL 1 M MgSO_4_, H_2_O to 1 L autoclaved) and collected by the modified Baermann funnel method using sterile beakers and tissue papers. The nematodes were transferred to capped, sterile 10 mL centrifuge tubes at room temperature and washed 5 times with M9 buffer to remove the *E*. *coli* OP50 from the nematode cuticle. The worms were then transferred to capped, sterile 5 mL centrifuge tubes containing 3 mL M9 buffer and they should be starved for 24 h at 20°C. For the feeding preferences experiment, nematodes mixed with adult and juvenile worms were concentrated in the collection tubes to a density of 1500 nematodes per 20 μL in M9 buffer.

Healthy gravid 3-day-old adult nematodes were harvested by rinsing the surfaces of the Petri dishes with M9 buffer at room temperature and then transferred to 10 mL centrifuge tubes, using 2–3 mL M9 buffer per plate. M9 buffer was added to a total volume of 8 mL, and most of the bacteria were removed from worms after washing 3 times at 5000 g for 3 minutes at 4°C. Next, 6 mL sterile H_2_O and 2 mL bleach (mixed with 1:2 v/v of 5 M NaOH and 5% NaClO solution) were added to the tubes. The tubes were shaken for a few seconds per minute for a total of 8 minutes. The liquid was discarded after centrifugation at 5000 g for 3 minutes at 4°C. The sediment was then washed 4 times with M9 buffer. This process killed the gravid adult worms and released the eggs, which remained intact and surface-sterilized. The eggs were cultured in M9 buffer overnight at 20°C for hatching, and the hatched larvae were transferred to freshly prepared NGM plates, which had been seeded with one of the five bacteria (Table 1). The dishes were then placed in a dark environment at 20°C for the nematodes rearing. Each week, worms were transferred to newly prepared NGM plates with the corresponding bacterium to ensure that the nematodes and their offspring could stay under the same and fresh conditions. These nematodes were prepared to use for the lifespan and brood size assays.

### Feeding preferences experiment

**Fig 1:**
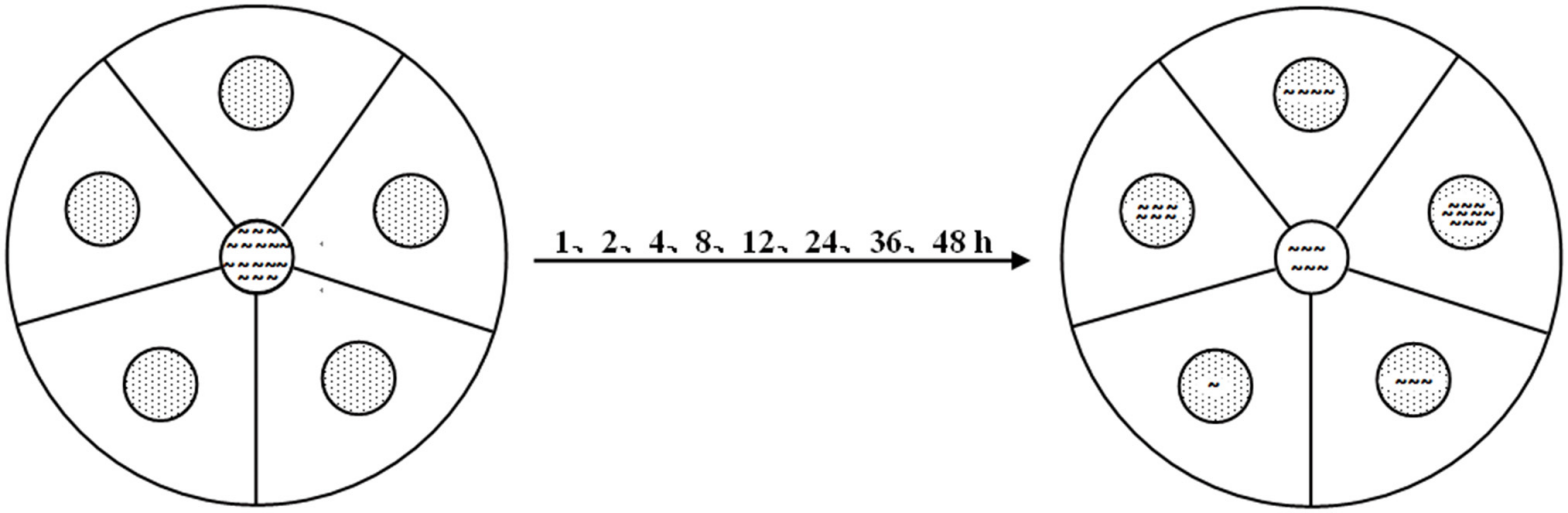
Feeding preferences experiment. At time point 0, nematodes were placed in the center of the dish and the bacteria were placed on the surrounding circles; the dishes were then stored in the dark at 20°C. Over time, the worms moved away from the center and toward the bacterial zone (indicated by the five circles with dots) and non-bacterial zone (the remainder of the dish). The worms in the bacterial and non-bacterial zones were counted at 1, 2, 4, 8, 12, 24, 36, 48 hours.

This experiment was conducted to determine the preferences of *C*. *elegans* for different bacteria, using 90 mm diameter Petri dishes filled with NGM. Each dish was divided into five equal sections containing a 15-mm diameter dot-circle that was 20 mm from a center circle where the worms were placed (Fig 1). In each section, the dot-circle was defined as the ‘bacterial zone’, and the remainder of the section was defined as the ‘non-bacterial zone’. The five bacterial suspensions (20 μL) were randomly placed on the bacterial zone respectively. Approximate 1500 individual nematodes were placed on the center circle, with 10 replicates. All dishes were placed at random in a dark incubator at 20°C. The number of nematodes that migrated to each bacterial zone and non-bacterial zone was recorded at 1, 2, 4, 8, 12, 24, 36, and 48 hours under a stereomicroscope at 50 × magnification.

### Bacterial respiration experiment

Liquid NGM medium was autoclaved, and 25 mL of this medium was transferred to the sterilized 130 mL saline bottles. The bacteria (100 μL) with OD_600_ values normalized to 1 were added to the liquid NGM, and then the bottles were sealed with soft air-tight lids and cultured in the dark at 20°C. The CO_2_ concentration was measured at 1, 2, 4, 8, 12, 24, 36, and 48 hours by gas chromatography (GC, Agilent 7890A, USA).

### Bacterial growth rate experiment

Liquid NGM medium was autoclaved and 50 mL of the medium was transferred to the sterilized 150 mL triangular flasks. The bacteria (500 μL) with OD_600_ values normalized to 1 were added to these triangular flasks, with 6 replicates per strain. Then, the flasks were cultured in the dark at 20°C with a shaking speed of 180 rpm. We counted the number of colony-forming units (CFUs) at each hour for the first 8 hours and then every two hours up to 48 hours. Each sample was diluted to a suitable concentration, and 100 μL of the diluted samples was spread onto an LB agar plate, followed by incubation at 30°C for 12–18 hours. The number of colonies formed was counted. To compare the growth rates of the different bacteria, we calculated the doubling time as $\text{G}=\frac{\left(t2-t1\right)}{(\text{lg}W2-\text{lg}W1)/\text{lg}2}$ where t1 and t2 represent two different times during the mid-exponential phase and W1 and W2 represent the number of colonies at t1 and t2, respectively [21].

### Nematode lifespan experiment

The survival analysis of worms cultured on the different tested bacteria was carried out at 20°C. On day 0, ten L1 stage nematodes (P0) were transferred to their corresponding NGM medium plates with fresh bacteria. Ten replicates were performed for each different bacterial strain. Surviving P_0_ worms were counted and transferred to fresh dishes daily until all of the worms had died. A worm was considered dead when it ceased moving and did not respond to a gentle touch with a platinum wire.

### Nematode brood size experiment

The brood size analysis of worms cultured on the different bacteria was performed in the analog condition with the survival analysis described above. One L4 stage nematode was randomly selected from each bacterial plate (NGM medium) and placed to a fresh plate with the corresponding bacterium as food. Ten replicates per strain were used. The worms were transferred to fresh dishes daily until they stopped laying eggs, and the number of eggs was recorded every day. The nematode brood size was determined based on the sum of total eggs laid by individual hermaphrodites.

### Statistical analysis

One-way analysis of variance (ANOVA) was used to analyze the following items: (1) the numbers of nematodes remaining in the bacterial and non-bacterial zones at each time point, (2) the bacterial growth rate and CO_2_ concentration released by bacterial respiration, and (3) the brood size and lifespan of *C*. *elegans* cultured with the five different bacteria. Duncan’s test (*p* < 0.05) was used to assess significant differences among the means. All statistical analysis was conducted using SPSS 16.0.

## Results

### Feeding preferences

**Fig 2:**
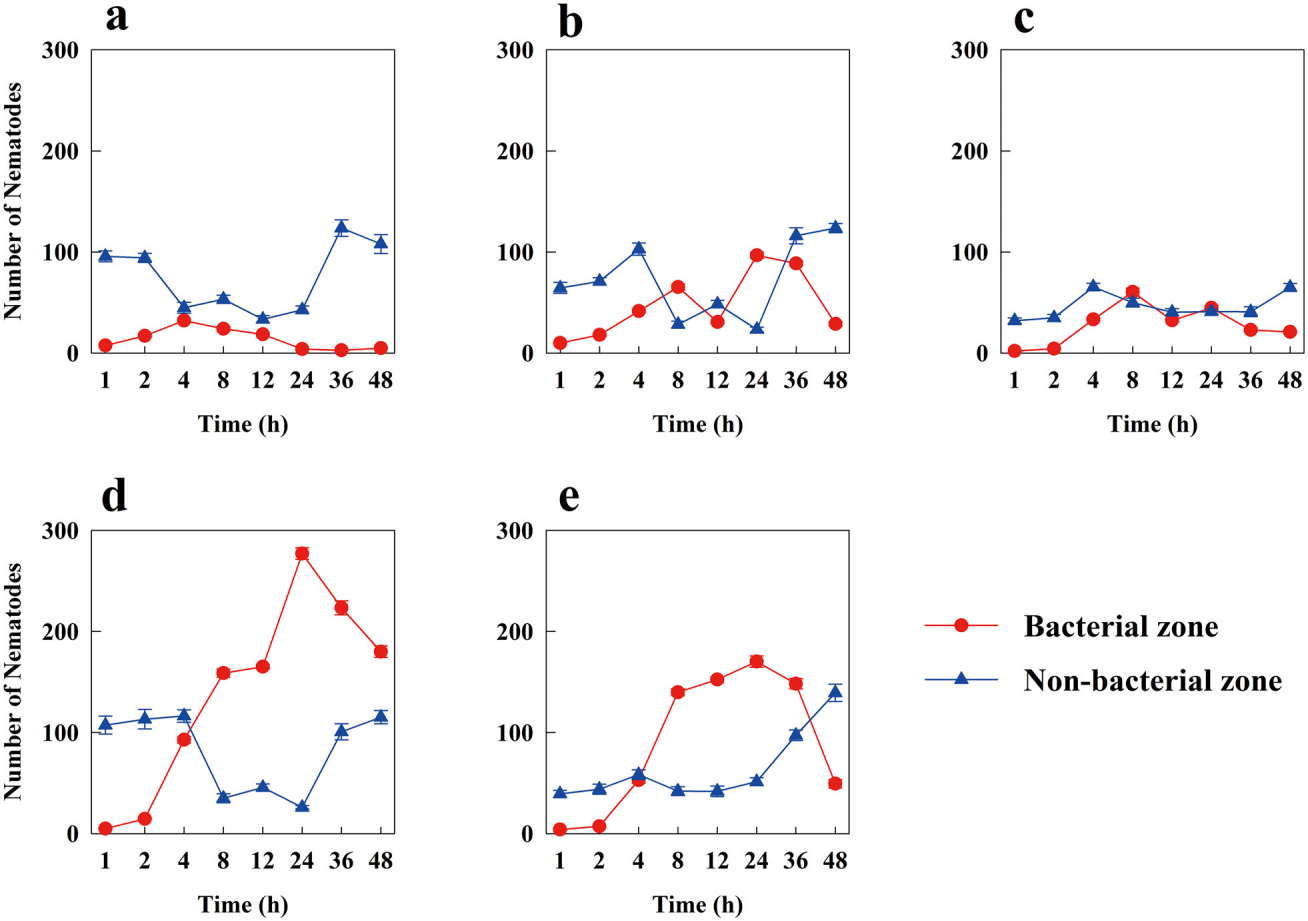
Numbers of *C*. *elegans* in the bacterial zone and non-bacterial zone with different bacteria. a, b, c, d, and e represent *B*. *amyloliquefaciens* JX1, *Variovorax* sp. JX14, *B*. *megaterium* JX15, *P*. *fluorescens* Y1 and *E*. *coli* OP50, respectively. Error bars represent standard errors.

*C*. *elegans* exhibited pronounced feeding preferences in our experiment. The number of *C*. *elegans* that moved to the five bacterial zones increased initially, particularly toward the *P*. *fluorescens* and *E*. *coli* lawns. However, the number of nematodes on the *B*. *amyloliquefaciens* and *B*. *megaterium* lawns decreased at 4 h and 8 h, respectively. The number of *C*. *elegans* remaining in the bacterial and non-bacterial zones showed an opposite trend after 4 hours (Fig 2), with more *C*. *elegans* in the bacterial zone than in the non-bacterial zone (Fig 2).

**Fig 3:**
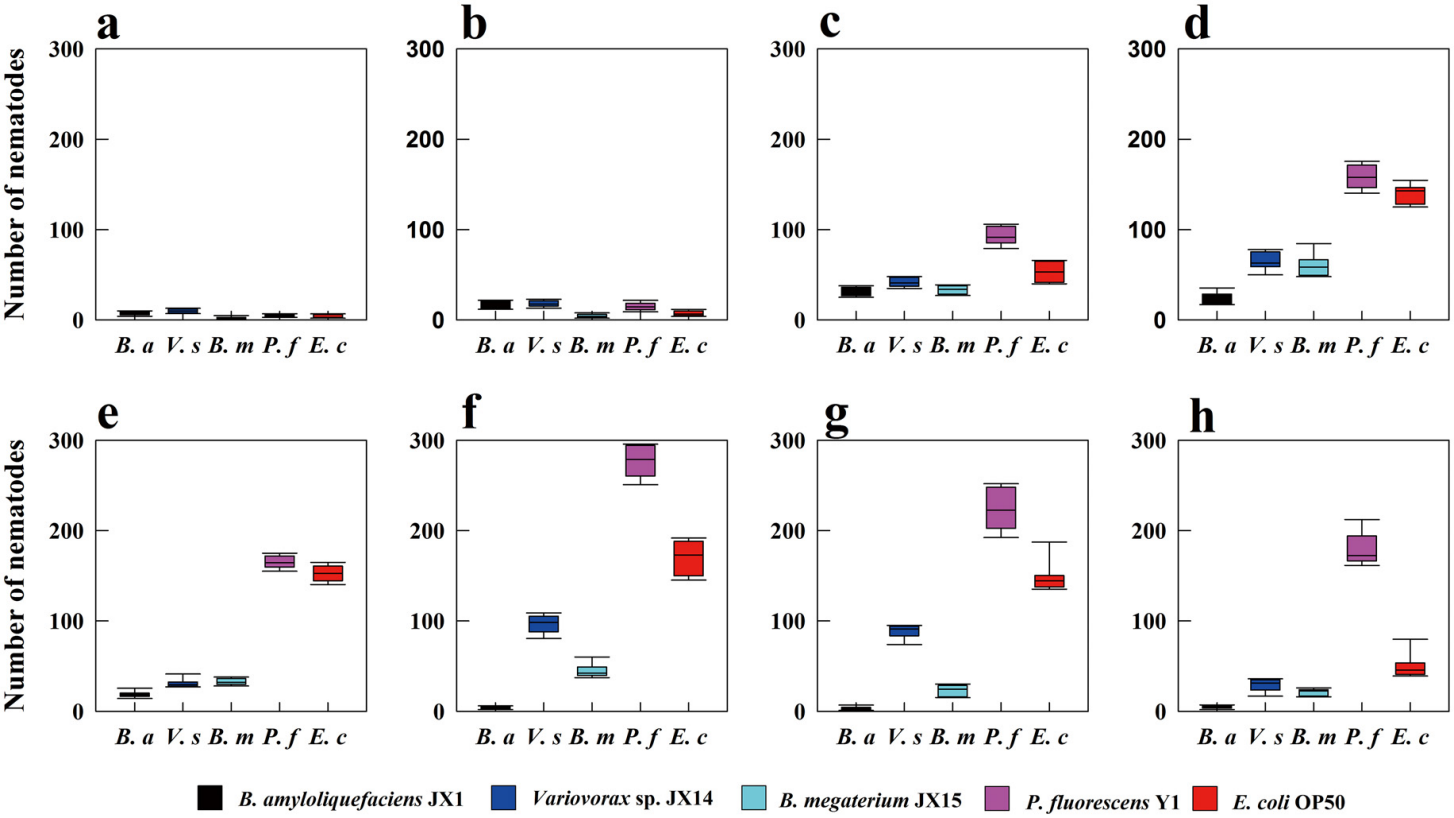
Variations in the number of *C*. *elegans* in the bacterial zone at different time points. Results are shown as box plots. a, b, c, d, e, f, g, and h represent 1, 2, 4, 8, 12, 24, 36, and 48 hours, respectively. *B*. *a*, *V*. *s*, *B*. *m*, *P*. *f*, *E*. *c* denotes *B*. *amyloliquefaciens* JX1, *Variovorax* sp. JX14, *B*. *megaterium* JX15, *P*. *fluorescens* Y1 and *E*. *coli* OP50, respectively (these abbreviations used in the following figures show the same meaning).

At the first hour, the number of *C*. *elegans* moving to the *Variovorax* sp. lawn was significantly higher than the number of worms moving to the other four bacterial lawns (*p* < 0.05, Fig 3a). However, the nematodes changed their choices shortly thereafter, with *P fluorescens* becoming the most preferred bacteria of *C*. *elegans* at 4 h, followed closely by *E*. *coli*; both bacteria were preferred over *Variovorax* sp., followed by *B*. *megaterium* and *B*. *amyloliquefaciens* (Fig 3c). This trend continued until the end of the experiment (Fig 3h). At 24 h, we observed an increase in the number of nematodes moving to the *Variovorax* sp. lawn, but there were still far fewer worms on *Variovorax* sp. than on either *P*. *fluorescens* or *E*. *coli* (Fig 3f).

Overall, *C*. *elegans* exhibited a hierarchy of food preferences, with *P*. *fluorescens* being the most preferred isolate, followed by *E*. *coli*, *Variovorax* sp. and *B*. *megaterium*; *B*. *amyloliquefaciens* was the least preferred (Fig 3).

### Bacterial respiration

**Fig 4:**
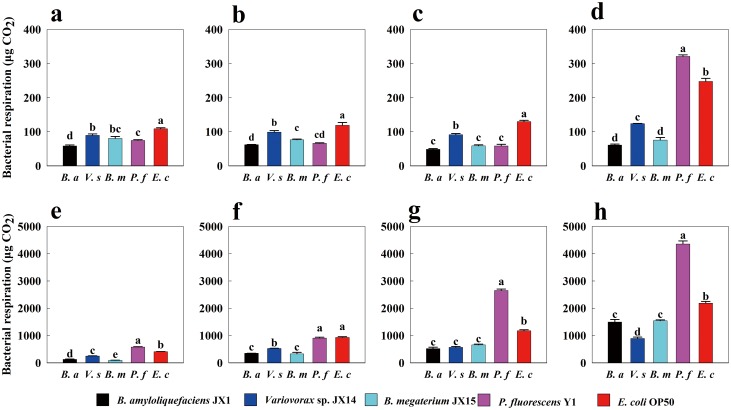
Bacterial respiration measured as CO_2_ released. Error bars represent standard errors, and letters over the error bars represent significant differences in bacterial respiration (*p*<0.05). a, b, c, d, e, f, g, and h represent 1, 2, 4, 8, 12, 24, 36, and 48 hours, respectively. The ordinate of Fig 4a–4d differs from the ordinate of Fig 4e–4h.

The CO_2_ concentrations released by the five bacteria were significantly different (*p* < 0.05, Fig 4). In the first 4 hours, *E*. *coli* produced the highest concentration of CO_2_ (Fig 4a–4c). Thereafter, the respiration of *P*. *fluorescens* was significantly (*p* < 0.05) higher than that of *E*. *coli*, followed by *Variovorax* sp., *B*. *amyloliquefaciens* and *B*. *megaterium* (Fig 4d). Over time, the bacteria produced increasing amounts of CO_2_ (Fig 4e–4h). From 8 hours to 48 hours, *P*. *fluorescens* released significantly more CO_2_ than the other four bacteria, except at 24 hours (Fig 4d–4h). We also used a Clark-type electrode (Hansatech, UK) to measure the respiration of the bacteria on vented solid NGM plates resuspended in 1 mL of sterilized water. There were no significant differences observed between these two methods (data not shown).

### Bacterial growth rates

We found that *P*. *fluorescens* had the fastest growth rate, and *B*. *megaterium* had the slowest (Table 2). There was a strong correlation between bacterial respiration and bacterial growth rates (Table 2 and Fig 4). Considering the respiration and growth rates of the bacteria, we defined those bacteria with relatively high respiration rates and rapid growth as active bacteria and those with relatively weak respiration and slow growth as inactive bacteria. Accordingly, *P*. *fluorescens* and *E*. *coli* were categorized as active bacteria, whereas *B*. *amyloliquefaciens* and *B*. *megaterium* were categorized as inactive bacteria.

**Table 2:** Bacterial growth rates.

Bacteria	Doubling time (h)
*B*. *amyloliquefaciens* JX1	1.78±0.10b
*Variovorax* sp. JX14	1.35±0.08b
*B*. *megaterium* JX15	2.44±0.29a
*P*. *fluorescens* Y1	0.42±0.00c
*E*. *coli* OP50	1.44±0.03b

Values are the means of the bacterial doubling time ± standard error.

Values with the same letter within a column indicate no significant difference (*p*>0.05).

### Nematode lifespan and brood size

**Fig 5:**
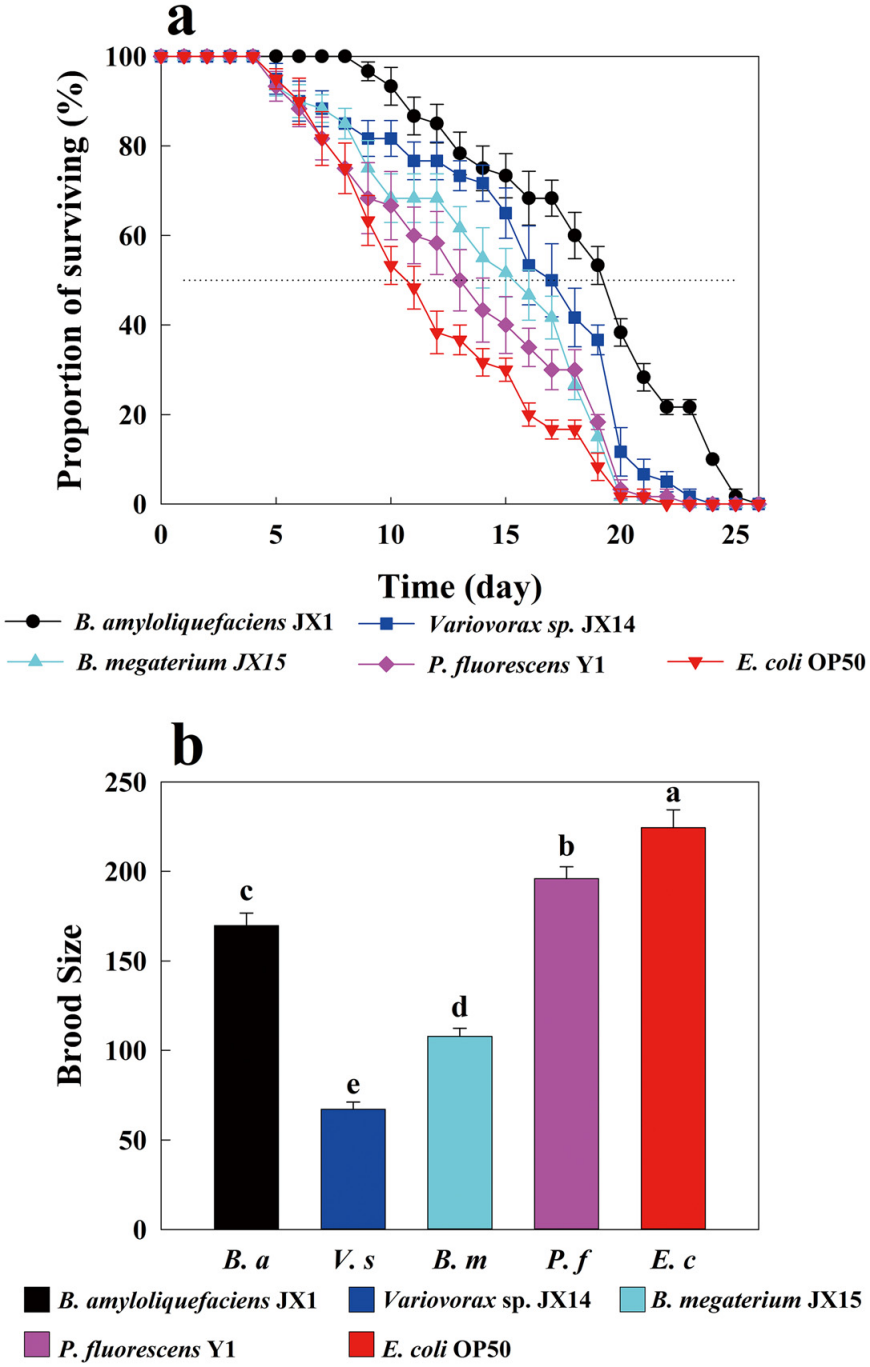
Effects of different bacteria on lifespan and brood size of *C*. *elegans*. (a) A survival curve showing the proportion of surviving worms in different bacterial environments at different times. Error bars represent standard errors. (b) Brood size indicates the ability of *C*. *elegans* to produce offspring in association with different bacterial isolates. Error bars represent standard errors, and letters over error bars represent significant differences in brood size.

Compared to *E*. *coli*, all the other four soil bacteria were found to prolong the lifespan of *C*. *elegans* (Fig 5a). Nematode lifespan is generally measured as the time to death for 50% of the worms [18, 22, 23]. The bacteria that most increased the lifespan of *C*. *elegans* was *B*. *amyloliquefaciens*, increasing it up to 80% (*p* < 0.01), followed by *Variovorax* sp., *B*. *megaterium*, and *P*. *fluorescens* (Fig 5a). In contrast to their effects on lifespan, these four soil bacteria strongly decreased the brood size of *C*. *elegans* relative to *E*. *coli* (Fig 5b). Moreover, the impacts of these four soil bacteria on worm brood size were significantly different (*p* < 0.05, Fig 5b). Except for *B*. *amyloliquefaciens*, the bacteria had seemingly opposite effects on *C*. *elegans* lifespan and brood size, extending the former but decreasing the latter.

## Discussion

BFNs consume a diverse variety of bacteria in soils, regulating the community, population, activity and phenotype of bacteria [24–29]. In turn, bacteria also affect the feeding behavior, lifespan and brood size of BFNs [19, 30–32].

It is possible that the bacterial cells or their metabolism release a food signal that attracts the worms [33]. In the feeding preferences experiment, we found that *C*. *elegans* exhibited marked food preferences for *P*. *fluorescens* Y1 and *E*. *coli* OP50 (Figs 2 and 3). When starved *C*. *elegans* encountered the five different bacteria, they initially showed random movement, presumably because they needed time to make decisions [34]. Subsequently, most of the worms moved to *P*. *fluorescens* Y1, which grew the fastest and emitted the highest concentration of carbon dioxide of the bacteria tested (Table 2, Figs 3 and 4). Thus, the CO_2_ gas released by bacterial respiration may be the food signal that attracted the starved *C*. *elegans*. Dusenbery [35] proposed that CO_2_ in basic solutions attracts *C*. *elegans*. Rasmann et al. [36] also suggested that CO_2_ is universally used as a cue to attract nematodes. However, other studies have demonstrated that *C*. *elegans* displays acute avoidance of CO_2_ under some conditions [37, 38]. These studies have also acknowledged that starved nematodes exhibit reduced CO_2_ avoidance [38], because *C*. *elegans* olfactory discrimination and acuity are enhanced during starvation [39]. Higher CO_2_ concentrations could indicate a larger and fresher bacterial population, an interpretation consistent with the results of our bacterial growth rate experiment (Table 2 and Fig 4); as a result, active bacteria, which release more CO_2_ and offer more food than inactive bacteria, attract *C*. *elegans*. Based on these ideas, we postulated that bacterial activity has significant effects on the feeding preferences of starved nematodes (Table 2, Figs 3 and 4). Additionally, gram-negative bacteria were much preferred by *C*. *elegans* over gram-positive bacteria (Figs 2 and 3). This result is consistent with previous studies showing that soil bacterial-feeding organisms prefer gram-negative bacteria over gram-positive bacteria [5, 40, 41].

However, nematodes feeding on the preferred, highly active bacteria *P*. *fluorescens* Y1 and *E*. *coli* OP50 had significantly shorter lifespan than those feeding on the less preferred, weakly active bacteria (Fig 5a). This suggests that bacterial activity may be harmful to BFNs. Bacterial activity affects bacterial colonization in the gut of nematodes, and bacterial colonization and proliferation in the intestine are considered to be major contributors to the death of nematodes [30]. Increases in CO_2_ concentration released by bacterial respiration have been found to be accompanied by decreases in O_2_ concentration [42]. Bacteria living around BFNs or within the BFNs intestine compete with BFNs for O_2_ and utilize resources within the BFNs intestine. Therefore, bacterial respiration and population growth in the nematodes gut may significantly affect nematodes lifespan. Portal-Celhay et al. [32] proposed that an intensively negative relationship between the lifespan of *C*. *elegans* and bacterial populations exists in the nematode’s intestine. The lifespan of *C*. *elegans* can be significantly increased by a diet lacking coenzyme Q [12, 43]. Similarly, Gomez et al. [19] reported that the longevity of *C*. *elegans* fed diets of respiratory deficient *E*. *coli* was dramatically enhanced compared to those fed *E*. *coli* OP50. Gomez et al. [19] also postulated that respiring *E*. *coli* utilize D-lactic acid and other metabolites in the nematodes guts as fuel for proliferation. In light of these studies, our findings suggest that *P*. *fluorescens* Y1 and *E*. *coli* OP50, both of which demonstrated high respiration and rapid reproductive rates, consumed more resources and metabolites in the intestine of nematodes than the other bacteria, thereby decreasing the lifespan of *C*. *elegans* (Table 2, Figs 4 and 5a). Thus, the most preferred food may not be the healthiest food for nematodes.

Compared to the active bacteria *P*. *fluorescens*Y1 and *E*. *coli* OP50, the inactive bacteria increased the lifespan of *C*. *elegans* (Fig 5a) but significantly decreased the brood size (Fig 5b). Our results also show a negative correlation between lifespan and brood size in *C*. *elegans* (Fig 5), which may be related to their life strategies. It is well known that r-strategist nematodes usually produce large numbers of eggs and have a short life cycle, whereas k-strategist nematodes have a low reproductive rate and a long life cycle [44–46]. Recently, more studies have focused on the relationship between lifespan and reproduction [47–49]. Partridge et al. [50] suggested that longevity and reproduction have a negative relationship with each other in model organisms. Barnes et al. [51] stated that increased reproduction was accompanied by a decreased lifespan in various organisms. Furthermore, dietary restriction, which is a popular study subject, might increase lifespan but inhibit reproduction in organisms [52]. When cultured with less favorable bacteria, nematodes ate fewer of them, increasing their lifespan but decreasing their reproduction, similar to dietary restriction. Food plays an important role in determining how reproduction affects lifespan [52]. In an unfavorable environment, organisms reduce their reproduction and increase their lifespan until the environment improves [53]. However, Salinas et al. [5] found that *Cephalobus brevicauda* had the lowest reproductive rate when fed its preferred bacteria. However, our brood size experiment demonstrated that *C*. *elegans* cultured with its preferred bacteria *P*. *fluorescens* Y1 and *E*. *coli* OP50 produced many more eggs than nematodes cultured with their less preferred bacteria (Figs 3 and 5). On low quality food, BFNs decrease their fecundity, employing resources reallocation to support lifespan processes [54]. In a less favorable bacterial environment, *C*. *elegans* decreased its brood size, allocating resources to support lifespan processes. This reallocation may be why less preferred bacteria extended the lifespan of *C*. *elegans* but decreased its brood size.

In conclusion, bacterial activity, including bacterial growth rate and bacterial respiration, directly and positively affect the feeding preferences and brood size of *C*. *elegans*, but they negatively influence the worms’ lifespan. The most preferred food may not be the most beneficial food for *C*. *elegans*, and therefore, worms may need to balance their consumption of preferred vs. beneficial bacteria.
